# PEth Cut‐Off Thresholds for Hazardous Alcohol Consumption Based on a Drinking Study

**DOI:** 10.1002/dta.3970

**Published:** 2025-11-18

**Authors:** Lisa Walther, Joanna Stenton, Therese Hansson, Anders Blomgren, Anders Andersson, Anders Isaksson

**Affiliations:** ^1^ Division of Clinical Chemistry and Pharmacology, Department of Laboratory Medicine Lund University Lund Sweden; ^2^ Department of Clinical Chemistry and Pharmacology Skåne University Hospital Lund Sweden

**Keywords:** alcohol markers, drinking study, ethanol, PEth, phosphatidylethanol

## Abstract

This study aimed to explore how PEth and other commonly used alcohol biomarkers (CDT, AST, ALT, and GGT) respond to regular consumption of what has been generally considered to correspond to low to moderate amounts of alcohol over a 2‐week period. A total of 21 voluntary participants (aged 31–69 years) took part in a 2‐week drinking study. Group 1 (*n* = 11) consumed one glass of wine daily (16 g of alcohol), close to the present Swedish limit for hazardous alcohol consumption, while Group 2 (*n* = 10) consumed two glasses daily (32 g of alcohol). Alcohol biomarkers were measured at baseline and at three further occasions during the study. After 1 week of alcohol consumption, all participants had measurable concentrations (> 0.005 μmol/L, ≈3.5 ng/mL) of both PEth‐homologues (PEth 16:0/18:1 and PEth 16:0/18:2). After 1‐ and 2‐week periods, significant differences in PEth levels were observed between Group 1 and Group 2. The correlation between the two PEth‐homologues was strong and increased as the study progressed. In contrast, other biomarkers showed little to no change during the study period. Both PEth‐homologues appear capable of identifying hazardous alcohol consumption. The current Swedish reporting threshold for PEth 16:0/18:1 (0.05 μmol/L, ≈35 ng/mL) demonstrates high specificity but low sensitivity in identifying hazardous alcohol consumption involving regular/daily intake. The sensitivity of the other biomarkers is insufficient for detecting alcohol consumption at this level.

## Graphical abstract

PEth 16:0/18:1 and 16:0/18:2 concentrations in voluntary participants taking part in a 2‐week drinking study with intake of one or two glasses of wine (16 or 32 g of ethanol) per day.

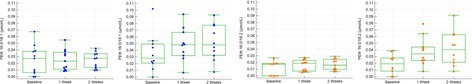

## 1 Introduction

Alcohol use disorders are a significant, yet often overlooked, cause of death, disease, and injury [1, 2]. These disorders also contribute to considerable social and economic burdens on individuals and society [1]. Consequently, accurate assessment of alcohol consumption is of crucial importance. Typically, alcohol intake data is based on self‐reported consumption gathered through interviews or questionnaires, such as the Alcohol Use Disorders Identification Test (AUDIT), the short form AUDIT‐C, and the Timeline Follow‐Back method [3, 4, 5, 6, 7, 8]. However, the reliability of these reports is frequently questioned [9, 10, 11, 12, 13]. Alcohol biomarkers can serve as an objective tool for diagnosis, monitoring, follow‐up, and treatment evaluation, either alone or in combination with self‐reported consumption data [14, 15, 16]. Commonly used alcohol markers, such as carbohydrate‐deficient transferrin (CDT), *γ*‐glutamyl transferase (GGT), aspartate aminotransferase (AST), and alanine aminotransferase (ALT), have primarily been used to detect heavy alcohol consumption or organ damage. However, their clinical sensitivity is insufficient for accurately identifying alcohol consumption at lower levels, limiting their effectiveness in such populations [17, 18, 19, 20].

Phosphatidylethanol (PEth) is an alcohol biomarker with high sensitivity and absolute specificity that has primarily been used in populations with moderate to heavy alcohol consumption [21, 22, 23]. Gradually, it has also been explored in groups with lower consumption levels [24, 25, 26, 27, 28]. PEth is a group of glycerophospholipid homologues formed in erythrocytes only when ethanol is present in the blood [29]. The two most common forms are PEth 16:0/18:1, which contains one palmitic and one oleic acid residue, and PEth 16:0/18:2, which contains one palmitic and one linoleic acid residue [30, 31]. PEth concentrations correlate with reported alcohol consumption, although the concordance between the two has varied considerably across studies [22, 24, 32, 33, 34, 35], making it difficult to estimate alcohol consumption from a PEth value. PEth 16:0/18:1 can, considering a half‐life of approximately 1 week [33, 36], be detected for several weeks depending on the amount of alcohol consumed and the sensitivity of the analytical methods used [21, 22, 36, 37, 38, 39].

This study seeks to explore PEth concentrations and compare them with older but still widely used alcohol biomarkers with a long window of detection in a 2‐week investigation involving daily alcohol intake at consumption levels that meet or exceed the present Swedish threshold for what is deemed hazardous alcohol use [40].

## 2 Material and Methods

### 2.1 Study Design and Participants

The study included 21 participants (10 men and 11 women), all of whom were low to moderate alcohol consumers. The inclusion criteria required participants to be between the ages of 30 and 70 and have no history of alcohol abuse. All individuals completed the AUDIT prior to participation, with inclusion requiring a score of ≤ 8 to exclude high alcohol consumers. To investigate the effects of low alcohol consumption, participants were asked to consume either one or two glasses of wine daily for 2 weeks. They were instructed not to consume alcohol 1 week before and 1 week after the 2‐week alcohol consumption period. Blood samples for the determination of PEth 16:0/18:1, PEth 16:0/18:2, CDT, GGT, AST, and ALT were collected in the morning on the first (Day 1, baseline) and second day (Day 2, after first intake) of the study, as well as after 1 and 2 weeks of alcohol consumption. Samples on Day 2 as well as after 1 and 2 weeks were drawn approximately 12 h after the latest intake of alcohol. An additional blood sample for PEth was collected 2–4 days after the 2‐week alcohol consumption period. PEth samples were immediately aliquoted and stored at −80°C until analysis to ensure stability [41]. Samples from the same participant were analyzed within the same analytical run to avoid between‐run variability.

The participants were divided into two groups. Group 1 consumed one glass of wine per day, while Group 2 consumed two glasses daily. One glass of wine was defined as 1.5 dL with an alcohol concentration of 13.5–14.5% v/v, providing 16–16.5 g of alcohol in Group 1 and 32–33 g in Group 2. These levels of consumption meet or exceed the present Swedish threshold for hazardous alcohol use (120 g ethanol per week ≈ 17 g/day) [40]. Participants were provided with wine and instructed to consume it in the evening. When necessary, participants were permitted to replace the wine with other wines at dinner outside of their homes. One participant in Group 1, who disliked wine, was allowed to substitute the wine with beer containing the equivalent amount of alcohol.

### 2.2 Determination of PEth 16:0/18:1 and PEth 16:0/18:2

PEth 16:0/18:1 and PEth 16:0/18:2 concentrations were determined using a liquid chromatography–tandem mass spectrometry (LC–MS/MS) method, as previously described [41]. Calibrators with concentrations of 0.005, 0.010, 0.050, 0.200, and 0.500 μmol PEth 16:0/18:1 (Enzo Life Sciences) and PEth 16:0/18:2 (Avanti Polar Lipids) were prepared in PEth‐free blood. For analysis, 200 μL of whole blood (unknown samples, calibrators, and control samples) was added to 1.2 mL of isopropanol containing 0.4‐μmol/L deuterium‐labeled PEth (PEth 16:0 (d31)/18:1, Avanti Polar Lipids). The mixture was centrifuged at 1500 g for 10 min, and the supernatant was transferred to a new tube and evaporated. The residue was dissolved in 200 μL of methanol/isopropanol (30/70) and transferred to a glass vial. Ten microliters of the solution was injected onto the column. The limit of quantification for both PEth homologues was 0.005 μmol/L. Control samples, derived from pooled patient material, showed coefficients of variation (CV) of 2.4% at 0.045 μmol/L and 4.3% at 0.022 μmol/L for PEth 16:0/18:1 and PEth 16:0/18:2, respectively.

### 2.3 Determination of CDT, GGT, AST, and ALT

CDT in serum was measured using an HPLC method and expressed as the percentage of disialotransferrin to total transferrin [42]. The coefficient of variation for the method was 3.8% and 3.4% at disialotransferrin levels of 1.3% and 2.3%, respectively. The upper reference limit for CDT in Sweden is 1.9% [42]. GGT, AST, and ALT were measured in plasma using the Cobas 6000 c501 (Roche Diagnostics, Mannheim, Germany). Reference values for liver enzymes were based on the Nordic reference interval project [43].

### 2.4 Statistical Evaluation

Descriptive statistics were calculated using Microsoft Excel (Microsoft Corp., Redmond, WA). Further statistical analyses were performed using SPSS for Windows (IBM Corp., Armonk, NY). Friedman's test was used for multiple comparisons, and the Wilcoxon signed‐rank test and Mann–Whitney *U* test were used for within‐group and between‐group comparisons, respectively. Statistical significance was set at a *p* value of < 0.05. Figures were generated using MedCalc statistical software (MedCalc Software Ltd., Ostend, Belgium).

### 2.5 Ethics

The study was approved by the Regional Ethics Review Board at Lund University (registry number 210/271), and written informed consent was obtained from all participants.

## 3 Results

All 21 participants completed the study. Descriptive statistics for demographic and anthropometric characteristics, AUDIT score, ethanol/kg bodyweight, alcohol markers, and liver enzymes are provided in Tables 1 and 2. Except for PEth (results detailed below), no statistically significant differences were observed between Group 1 and Group 2 for the variables listed in Table 2 during the 2‐week alcohol consumption period. Similarly, no significant differences were found between men and women for any of the variables at any time point.

**TABLE 1:** Descriptive statistics of demographic and anthropometric characteristics, AUDIT scores, and ethanol/kg bodyweight of the two groups, mean (median) range.

	Group 1 (*n* = 11)	Group 2 (*n* = 10)
Age (year)	55 (54) 38–66	52 (57) 31–69
Gender men (women)	5 (6)	5 (5)
AUDIT (score)	3.3 (3) 2–8	3.2 (3) 1–5
AUDIT‐C (score)	3.0 (3) 2–5	3.0 (3) 1–5
Weight (kg)	75 (77) 55–94	76 (75) 56–118
BMI (kg/m^2^)	25 (24) 20–32	25 (24) 20–34
Ethanol/kg bodyweight (g/kg)	0.22 (0.21) 0.17–0.29	0.43 (0.43) 0.27–0.57

**TABLE 2:** Descriptive statistics for alcohol markers and liver enzymes for the study participants, mean (median) range.

Group 1 (*n* = 11)	Day 1 (baseline)	Day 2	1 week	2 weeks
PEth 16:0/18:1 (μmol/L)^a^	−(0.027)	−(0.027)	0.025 (0.023)	0.025 (0.028)
	< 0.005–0.067	< 0.005–0.061	0.008–0.055	0.010–0.043
PEth 16:0/18:2 (μmol/L)^b^	−(0.017)	−(0.018)	0.016 (0.019)	0.017 (0.016)
	< 0.005–0.027	< 0.005–0.024	0.005–0.027	0.007–0.029
CDT (%)	1.1 (1.1)	1.1 (1.1)	1.1 (1.2)	1.1 (1.1)
	0.8–1.2	0.8–1.3	0.8–1.3	0.8–1.2
GGT (μkat/L)	0.54 (0.32)	0.52 (0.30)	0.58 (0.32)	0.51 (0.31)
	0.16–2.12	0.16–2.14	0.14–2.74	0.20–1.90
AST (μkat/L)	0.42 (0.42)	0.39 (0.36)	0.40 (0.38)	0.37 (0.37)
	0.31–0.68	0.28–0.65	0.24–0.61	0.25–0.46
ALT (μkat/L)	0.42 (0.39)	0.41 (0.37)	0.43 (0.40)	0.36 (0.38)
	0.14–0.99	0.14–0.86	0.10–1.00	0.09–0.48

Group 2 (*n* = 10)	Day 1 (baseline)	Day 2	1 week	2 weeks
PEth 16:0/18:1 (μmol/L)^a^	−(0.028)	−(0.037)	0.049 (0.048)	0.052 (0.048)
	< 0.005–0.102	< 0.005–0.105	0.007–0.094	0.008–0.093
PEth 16:0/18:2 (μmol/L)^b^	−(0.019)	−(0.024)	0.038 (0.034)	0.042 (0.040)
	< 0.005–0.039	< 0.005–0.043	0.008–0.079	0.008–0.092
CDT (%)	1.1 (1.1)	1.2 (1.2)	1.2 (1.2)	1.2 (1.2)
	0.8–1.4	0.8–1.4	0.8–1.5	0.8–1.7
GGT (μkat/L)	0.36 (0.30)	0.36 (0.30)	0.36 (0.29)	0.37 (0.32)
	0.15–0.68	0.15–0.68	0.20–0.62	0.20–0.60
AST (μkat/L)	0.49 (0.40)	0.47 (0.42)	0.44 (0.42)	0.43 (0.44)
	0.31–1.15	0.26–1.05	0.35–0.66	0.31–0.56
ALT (μkat/L)	0.55 (0.37)	0.53 (0.39)	0.48 (0.41)	0.48 (0.38)
	0.17–2.14	0.18–2.09	0.20–1.60	0.24–1.50

^a^ 1 μmol/L = 703 ng/mL.

^b^ 1 μmol/L = 701 ng/mL.

At baseline, significant correlations were found between both AUDIT and AUDIT‐C scores and the two PEth homologues, but not between AUDIT and any other alcohol marker. The highest correlations were observed with AUDIT (*rs* = 0.709, *p* = 0.000 for PEth 16:0/18:1 and *rs* = 0.516, *p* = 0.017 for PEth 16:0/18:2).

### 3.1 PEth 16:0/18:1 and PEth 16:0/18:2

**FIGURE 1:**
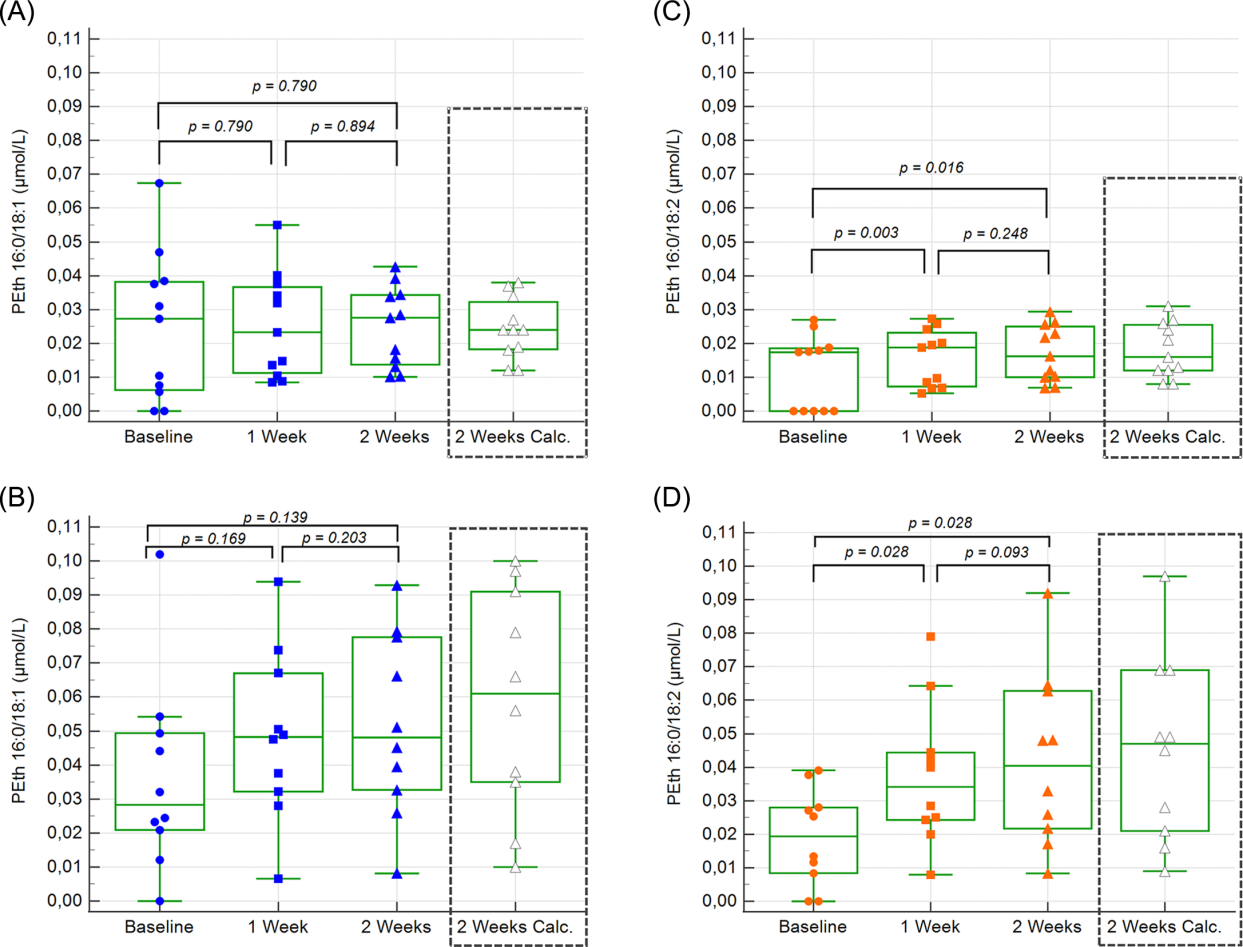
Box and whisker plot of the PEth homologues at baseline, 1 and 2 weeks of alcohol consumption for Groups 1 and 2. The fourth box and whisker plot shows calculated steady state concentrations (including correction for concentrations at baseline). (A) PEth 16:0/18:1 Group 1. (B) PEth 16:0/18:1 Group 2. (C) PEth 16:0/18:2 Group 1. (D) PEth 16:0/18:2 Group 2.

At baseline, 18 out of 21 participants had detectable PEth 16:0/18:1 concentrations (≥ 0.005 μmol/L, ≈3,5 ng/mL), compared to 14 participants for PEth 16:0/18:2 (Table 2). After the first day of alcohol consumption, the number of participants with detectable PEth increased to 19 for PEth 16:0/18:1 and 17 for PEth 16:0/18:2. After 1 week of alcohol consumption, all participants had detectable concentrations of both PEth homologues. Over the 2‐week study, PEth 16:0/18:1 increased in 12 participants (five in Group 1 and seven in Group 2) and decreased in eight (five in Group 1 and three in Group 2), while all but one participant (in Group 1) showed an increase in PEth 16:0/18:2. The distribution of PEth levels during the study for the two groups is shown in Table 2 and Figure 1. After 1 and 2 weeks of alcohol consumption, significant differences were observed between the two groups, with mean values approximately twice as high in Group 2 as in Group 1, while no differences were observed between the groups at baseline (Table 2).

A significant difference in PEth 16:0/18:2 levels was found between baseline (Day 1), Day 2, and after 1 and 2 weeks of alcohol consumption, as determined by Friedman's test (*p* < 0.000). Further testing with the Wilcoxon signed‐rank test showed significant increases in PEth 16:0/18:2 concentrations between baseline and 1 week (*p* = 0.001), baseline and 2 weeks (*p* = 0.001), and between 1 and 2 weeks (*p* = 0.042). However, there was no significant change in PEth 16:0/18:1 levels during the study (*p* = 0.185), either in Group 1 or Group 2.

No trend in PEth levels in either Group 1 or 2 was seen when considering dosing of ethanol per kg body weight likely due to dilution by other sources of variation.

**FIGURE 2:**
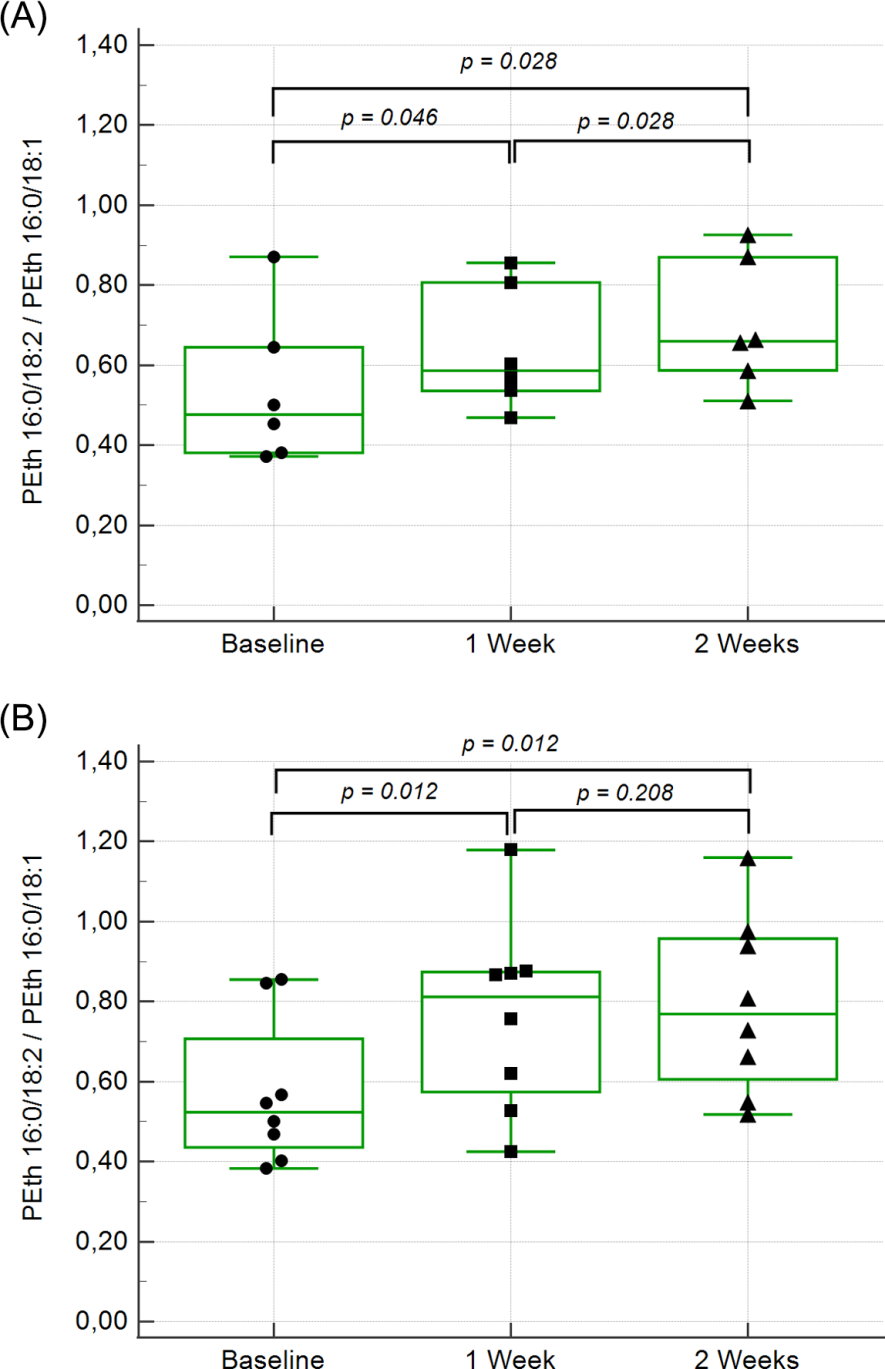
Box and whisker plot of the ratio between PEth 16:0/18:2 and PEth 16:0/18:1 at baseline, 1 and 2 weeks of alcohol consumption (*n* = 14). (A) Group 1. (B) Group 2.

The correlations between the two PEth homologues were highly significant and increased during the study: *rs* = 0.705 (*p* = 0.005, *n* = 14) at baseline and *rs* = 0.918 (*p* = 0.000, *n* = 21) after 2 weeks of alcohol consumption. The ratio of PEth 16:0/18:2 to PEth 16:0/18:1 changed significantly over the course of the study (Figure 2). All participants except one showed a decrease in PEth concentrations in samples taken 2–4 days after alcohol consumption ceased. The mean half‐lives for PEth 16:0/18:1 and PEth 16:0/18:2 were calculated to be 7.6 (median 6.8, range 4.0–12.8) and 4.8 (median 4.5, range 2.7–8.0) days, respectively. The participant with an increase in PEth in the post‐drinking sample was excluded from this calculation.

Several participants had significant concentrations of PEth at baseline. The contribution of the baseline values to the measured concentrations at 1 and 2 weeks was calculated using the individual half‐lives of the two PEth forms. For those individuals where individual half‐lives were missing (six for PEth 16:0/18:1 and seven for PEth 16:0/18:2), the group mean value was used. The results for PEth at 1 and 2 weeks could then be corrected using the calculated contribution from the baseline value (Table 3). After correction for baseline values, significant increases between 1 and 2 weeks were seen in both PEth 16:0/18:1 and PEth 16:0/18:2 (Table 3). Since the half‐lives of PEth 16:0/18:1 and PEth 16:0/18:2 were calculated to be 7.6 and 4.8 days, respectively, neither PEth form had reached complete dynamic equilibrium (steady state concentrations) either after 1 or 2 weeks. However, steady state concentrations could be calculated from baseline‐corrected values for both 1 and 2 weeks using the half‐lives given above (Table 3 and Figure 1). Calculated steady state concentrations based on values at 1 and 2 weeks were in good agreement (Table 3).

**TABLE 3:** Calculated results for PEth 16:0/18:1 and PEth 16:0/18:2, mean (median) range.

Group 1 (*n* = 11)	1 week BL^a^ (μmol/L)	2 weeks BL^a^ (μmol/L)	*p* *	1 week BL^a^ + DE^b^ (μmol/L)	2 weeks BL^a^ + DE^b^ (μmol/L)
PEth 16:0/18:1	0.011 (0.010) 0.006–0.019	0.017 (0.016) 0.008–0.026	0.003	0.023 (0.026) 0.012–0.037	0.024 (0.024) 0.012–0.038
PEth 16:0/18:2	0.011 (0.012) 0.005–0.018	0.015 (0.014) 0.007–0.027	0.003	0.019 (0.020) 0.008–0.029	0.018 (0.016) 0.008–0.031

Group 2 (*n* = 10)	1 week BL^a^	2 weeks BL^a^	*p* value*	1 week BL^a^ + DE^b^	2 weeks BL^a^ + DE^b^
PEth 16:0/18:1	0.032 (0.024) 0.006–0.064	0.044 (0.037) 0.008–0.084	0.005	0.060 (0.056) 0.012–0.121	0.059 (0.061) 0.010–0.100
PEth 16:0/18:2	0.032 (0.027) 0.008–0.071	0.040 (0.037) 0.008–0.089	0.005	0.048 (0.043) 0.012–0.105	0.045 (0.047) 0.009–0.097

^a^ BL = baseline corrected.

^b^ DE = dynamic equilibrium corrected.

* Difference between baseline corrected (BL) results at 1 and 2 weeks.

### 3.2 CDT

One female participant in Group 2 showed an increase in CDT from 1.3 at Day 2 to 1.7% at 2 weeks but with this exception no significant changes were observed between Day 2 and 1 week or Day 2 and 2 weeks.

### 3.3 GGT, AST, and ALT

All participants, except for two, had GGT, AST, and ALT levels within the reference range. One male participant experienced a slight increase in GGT, which returned to normal during the study, while another male participant had a slight increase in both AST and ALT at baseline. The elevated AST returned to normal during the study, and the ALT decreased, though it did not fully normalize.

## 4 Discussion

Accurate information on alcohol consumption is crucial in both clinical and research settings. Alcohol biomarkers provide an objective way to estimate alcohol consumption levels [9, 44, 45, 46]. As a metabolite of ethanol, PEth has the potential to detect and assess even low to moderate levels of alcohol consumption. This study assesses the alcohol marker PEth and compares its performance to other commonly used alcohol markers in an experimental setting, where participants voluntarily consumed low amounts of alcohol daily for 2 weeks.

Since the participants in this study were low or social drinkers, with AUDIT scores ranging from 1 to 8, the majority had detectable baseline levels of PEth, with concentrations both increasing and decreasing throughout the study. This probably reflects that some of the participants decreased, and some increased their average alcohol consumption during the study. At baseline, PEth 16:0/18:2 concentrations were lower than those of PEth 16:0/18:1, and all but one participant showed an increase in PEth 16:0/18:2 levels during the study. The ratio of PEth 16:0/18:2 to PEth 16:0/18:1 increased early in the study, suggesting a higher synthesis rate of PEth 16:0/18:2 compared to PEth 16:0/18:1. These findings align with results from a clinical study on PEth following a single alcohol intake [36] as well as an experimental in vitro study [47].

By the end of the first week, all participants had detectable levels of PEth, with mean (median) concentrations of 0.025 (0.023) μmol/L for PEth 16:0/18:1 in Group 1 and 0.049 (0.048) μmol/L in Group 2. Further increases between Week 1 and Week 2 were modest. A similar trend, though with slightly lower values, was seen for PEth 16:0/18:2, with mean concentrations of 0.016 μmol/L (median 0.019 μmol/L) in Group 1 and 0.038 μmol/L (median 0.034 μmol/L) in Group 2. While PEth levels overlapped between the two groups, significant differences were observed for both forms of PEth. After 2 weeks, the range of PEth values was wider in Group 2 (0.008–0.093 μmol/L) compared to Group 1 (0.010–0.043 μmol/L) (Table 2 and Figure 1). Baseline‐ and dynamic‐equilibrium corrected results were essentially identical to the measured values at 2 weeks (Tables 2 and 3 and Figure 1). Using published half‐life values for PEth [33] produced nearly identical calculated results. Support for the validity of the calculated results is also that the outcome was practically identical when baseline‐corrected values from either 1 or 2 weeks were used in the calculations (Table 3).

PEth indicates alcohol exposure rather than the precise quantity of alcohol consumed. Various factors, including dose, total body water, drinking patterns, whether alcohol is consumed with or without food, and the rates of PEth formation and degradation, can all influence PEth levels [33, 47, 48]. The participants in this study were not given specific instructions regarding how to consume the wine, such as how quickly to drink or whether to drink with food, although evening consumption was recommended. Drinking patterns may be more variable when consuming two glasses of wine rather than one. One participant in Group 2 with a remarkably low increase in PEth during the study reported consuming the wine very slowly during the evening, which likely resulted in both lower blood alcohol and PEth levels. In a separate study where participants consumed one or two glasses of wine daily for 3 months, the median PEth 16:0/18:1 concentration was 0.022 μmol/L [24]. As discussed in that study, it is challenging to follow a strict protocol, i.e., drinking a specified amount of alcohol for a certain period of time. The magnitude of the challenge would seem to increase with the length of the time period. However, we believe adherence to the protocol in the present 2‐week study was good, especially considering the short period and the low alcohol consumption involved. This may help explain the slightly higher mean (median) values observed in this study compared to the previous one, even though both studies involved similar amounts of regular (daily) alcohol intake.

Drinking patterns, for example, regular drinking versus binge drinking, will, however, affect the results for PEth, which can be calculated and shown using data taken from a previously published study [37]. In that study, 11 healthy volunteers consumed an amount of alcohol (Vodka, 40%) that was estimated to give rise to a maximum blood ethanol concentration of 1 g/kg on each of five consecutive days. The mean amount of alcohol consumed on each occasion was 77 g (range 50–109 g), with the total volume of Vodka (mean 242, range 155–340 mL), consumed within 1 h. This amount of ethanol corresponds to approximately five glasses of wine (16 g/glass). Thus, the total amount of ethanol consumed during 5 days (one glass per day) in our study was in this case consumed on a single occasion. This amount of ethanol was calculated to give rise to a mean PEth 16:0/18:1 concentration of 0.046 μmol/L (≈32 ng/mL) in the sample collected on the morning the day after intake. This is in line with results from two other studies on PEth after single intake of alcohol [49, 50]. With a half‐life of 1 week, this concentration will have decreased to about 0.023 μmol/L (≈16 ng/mL) after 1 week of abstinence, that is, similar to the PEth concentration (0.025 μmol/L, ≈17 ng/mL) found in our study after the daily consumption of one glass of wine. This suggests that for the same amount of alcohol consumed, binge drinking leads to higher PEth concentrations than regular drinking. However, the results are also influenced by the interval between consumption and the timing of blood sampling. Nevertheless, the fact that binge drinking causes a higher PEth value may even be appropriate, as binge drinking is regarded as more harmful [51].

At the end of the study, a final blood sample was collected 2–4 days after the cessation of alcohol consumption to examine PEth degradation. The calculated half‐lives of PEth 16:0/18:1 and PEth 16:0/18:2 were 7.6 and 4.8 days, respectively, which align with previously published results [33, 52]. Given the indicated half‐lives of PEth 16:0/18:1 and 16:0/18:2, the current 2‐week test period corresponds to 2–3 half‐lives and since most individuals had significant concentrations of PEth at baseline and PEth 16:0/18:1 did not show any significant difference during the study, the results for the two PEth homologues at 2 weeks would appear to represent steady state concentrations at the two consumption levels. This is further corroborated by calculations that consider both the baseline values and half‐lives of PEth. Furthermore, these findings are consistent with data from a short (3‐day) drinking study [53] and a study using smartphone‐recorded alcohol consumption over 14 days [54].

PEth at baseline showed a strong correlation with reported consumption according to AUDIT, with rs = 0.709, *p* = 0.000 for PEth 16:0/18:1 and rs = 0.516, *p* = 0.017 for PEth 16:0/18:2. This correlation is notably strong, considering the small interindividual differences in PEth levels and reported alcohol consumption in the present study group. This stands in stark contrast to a previous study on alcohol‐dependent individuals, where no correlation was found between PEth and reported consumption based on either AUDIT or TLFB [22]. The correlation between PEth and reported alcohol consumption has varied across studies [22, 24, 32, 33, 34, 35], indicating that self‐reported consumption may not be reliable and can differ significantly between studies and study groups. Despite the strong correlation between AUDIT and PEth in this study, the PEth levels measured before and after the period of standardized alcohol intake suggest that alcohol consumption as reported through AUDIT, may have been somewhat underreported even in the present study group.

In Sweden, PEth 16:0/18:1 is a widely used alcohol marker [29, 41, 55]. Swedish laboratories agree not to report PEth 16:0/18:1 results below 0.05 μmol/L (≈35 ng/mL) and to interpret values under this threshold as indicating no or low alcohol consumption [56]. Concentrations between 0.05 and 0.30 μmol/L (35–200 ng/mL) are interpreted as corresponding to moderate alcohol consumption, while values above 0.30 μmol/L suggest excessive consumption [56]. Internationally, other cut‐off values are used with < 20 ng/L (≈0.03 μmol/L) interpreted as compatible with abstinence or low alcohol consumption, while ≥ 20–200 and ≥ 200 ng/mL (≈0.30 μmol/L) are interpreted as alcohol consumption or strongly suggestive of chronic excessive alcohol consumption, respectively [57]. All these cut‐offs are based on clinical experience considering the analytical sensitivity of the methods used, rather than on established relationships between PEth levels and specific alcohol consumption data.

The definition of a standard drink and what constitutes hazardous alcohol consumption differs across countries [58, 59]. In Sweden, consuming more than 10 units per week (1 unit = 12 g ethanol) is considered hazardous alcohol use for both men and women [40]. This equates to about 17 g of ethanol per day. In our study, the mean PEth 16:0/18:1 values for Groups 1 and 2, who consumed 16 or 32 g of alcohol daily in 2 weeks, were 0.025 μmol/L (≈18 ng/mL) and 0.052 μmol/L (≈37 ng/mL), respectively. This response in PEth is significantly lower than that typically derived from self‐reported consumption data, indicating a high degree of underreporting.

After 2 weeks none of the participants in Group 1 had a PEth level exceeding 0.050 μmol/L (≈35 ng/mL) whether measured or calculated, whereas several participants had values exceeding 0.030 μmol/L (≈20 ng/mL)—four measured and three calculated (Figure 1). None in Group 2 who consumed twice the amount of alcohol had a PEth level above 0.100 μmol/L (≈70 ng/mL) whether measured or calculated (Figure 1). The Swedish reporting threshold for PEth 16:0/18:1 (0.050 μmol/L, ≈35 ng/mL) thus demonstrates high specificity but low sensitivity in identifying hazardous alcohol consumption according to the current Swedish definition. Depending on whether diagnostic specificity or sensitivity is prioritized, alternative cut‐offs or reporting thresholds could be considered. Since other consumption patterns, that is, different from regular (daily) alcohol intake, appear to yield somewhat higher PEth concentrations, the current threshold value for PEth 16:0/18:1 (0.05 μmol/L) still seems to represent a reasonable compromise between sensitivity and specificity. A corresponding cut‐off for PEth 16:0/18:2 would seem to be 0.03 μmol/L (≈20 ng/mL). Alternative definitions of hazardous alcohol consumption or special clinical situations, for example pregnancy [26], may warrant the use of different (lower or higher) cut‐off values or decision limits.

Regarding CDT, no significant changes were observed, except for one participant who showed a moderate increase from 1.3% at Day 2 to 1.7% at 2 weeks, still below the upper reference limit of 1.9%.

In this short study, regular low‐dose alcohol consumption did not affect liver enzymes at the group level. The two individuals with slight increases in liver enzymes at baseline, which normalized during the study, had no obvious explanation, though a previous study observed a reduction in liver lipid content in participants consuming low amounts of alcohol regularly [60].

## 5 Conclusion

Both PEth homologues can effectively detect low alcohol consumption. Although results were somewhat overlapping, PEth values were significantly higher in Group 2 (double the alcohol intake) compared to Group 1, with approximately twice as high mean values in Group 2. Other factors, such as total body water, consumption patterns, whether alcohol is consumed with or without food and the rates of PEth formation and degradation, can also influence PEth levels. However, the current reporting threshold for PEth 16:0/18:1 (0.050 μmol/L, ≈35 ng/mL) demonstrates high specificity but low sensitivity for identifying hazardous alcohol consumption involving regular (daily) intake. Alternative definitions of hazardous alcohol consumption or special clinical situations, for example pregnancy, may warrant the use of different (lower or higher) cut‐off values or decision limits. A slightly lower threshold would seem to be appropriate for PEth 16:0/18:2. Other markers, such as CDT and liver enzymes, showed no or minimal changes during the drinking study, making them ineffective as alcohol markers at low levels of consumption.

## Conflicts of Interest

The authors declare no conflicts of interest.

## Data Availability

The data that support the findings of this study are available from the corresponding author upon reasonable request.
